# Clinical Efficacy of Novel Patient-Covering Negative-Pressure Box for Shielding Virus Transmission during Esophagogastroduodenoscopy: A Prospective Observational Study

**DOI:** 10.3390/diagnostics11091679

**Published:** 2021-09-14

**Authors:** Shintaro Fujihara, Hideki Kobara, Noriko Nishiyama, Naoya Tada, Kazuhiro Kozuka, Takanori Matsui, Taiga Chiyo, Nobuya Kobayashi, Tingting Shi, Tatsuo Yachida, Toshio Uchida, Taichi Nagatomi, Haruo Oba, Tsutomu Masaki

**Affiliations:** 1 Department of Gastroenterology and Neurology, Faculty of Medicine, Kagawa University, Kagawa 761-0793, Japan; joshin@med.kagawa-u.ac.jp (S.F.); n-nori@med.kagawa-u.ac.jp (N.N.); n-tada@med.kagawa-u.ac.jp (N.T.); koduka2525@gmail.com (K.K.); tk-matsui@med.kagawa-u.ac.jp (T.M.); t_chiyo@med.kagawa-u.ac.jp (T.C.); nobuyak@med.kagawa-u.ac.jp (N.K.); shitingtingc@med.kagawa-u.ac.jp (T.S.); tyachida@med.kagawa-u.ac.jp (T.Y.); tmasaki@med.kagawa-u.ac.jp (T.M.); 2 Department of Gastroenterology, Kagawa Prefectural Shirotori Hospital, Kagawa University, Kagawa 769-2788, Japan; 3 Center for Industrial-Academic Partnership and Intellectual Property, Kagawa University, Kagawa 760-8521, Japan; uchida@eng.kagawa-u.ac.jp (T.U.); nagatomi.taichi@kagawa-u.ac.jp (T.N.); 4 Department of Engineering and Design, Kagawa University, Kagawa 760-8521, Japan; oba.haruo@kagawa-u.ac.jp

**Keywords:** esophagogastroduodenoscopy, aerosol, virus transmission, COVID-19

## Abstract

Esophagogastroduodenoscopy (EGD) has a high risk of virus transmission during the current coronavirus disease 2019 era, and preventive measures are under investigation. We investigated the effectiveness of a newly developed patient-covering negative-pressure box system (Endo barrier^®^) (EB) for EGD. Eighty consecutive unsedated patients who underwent screening EGD with EB use were prospectively enrolled. To examine the aerosol ratio before, during, and after EGD, 0.3- and 0.5-μm aerosols were measured every 60 s using an optical counter. Moreover, the degree of contamination of the examiners’ goggles and vinyl gowns was assessed before and after EGD using a rapid adenosine triphosphate (ATP) test for simulated droplets. Data were available in 73 patients and showed that 0.3- and 0.5-μm particles did not increase in 95.8% (70/73) and 94.5% (69/73) of patients during EGD under EB. There were no significant differences in the total 0.3- or 0.5-μm particle counts before versus after EGD. The difference in the ATP levels before and after EGD was −0.6 ± 16.6 relative light units (RLU) on goggles and 1.59 ± 19.9 RLU on gowns (both within the cutoff value). EB use during EGD may provide a certain preventive effect against aerosols and droplets, decreasing examiners’ exposure to viruses.

## 1. Introduction

Coronavirus disease 2019 (COVID-19) is a global pandemic. The COVID-19 pandemic has continued to cause dramatic shifts in the practice of endoscopy. In particular, the COVID-19 pandemic has had an extraordinary impact on the delivery of gastrointestinal endoscopy, with an initial reduction to 12% of the prepandemic level in the United Kingdom [1]. This reduction in activity has the serious effect of delaying the diagnosis of important diseases such as early gastrointestinal malignancies [1]. Esophagogastroduodenoscopy (EGD) is an aerosol-generating procedure that has the potential to transmit the virus from infected patients to health care workers (HCWs); in fact, among all HCWs in the field of gastroenterology, the risk of COVID-19 is reportedly highest among endoscopy technicians [2]. Several guidelines, including those of the World Endoscopy Organization, the European Society of Gastrointestinal Endoscopy, the Asian Pacific Society of Digestive Endoscopy, and the Japan Society of Gastrointestinal Endoscopy, recommend wearing standard or full personal protective equipment (PPE) during gastrointestinal endoscopic procedures to minimize the risk of infection to HCWs during the pandemic [3,4,5,6].

Severe acute respiratory syndrome coronavirus 2 (SARS-CoV-2), the virus that causes COVID-19, is highly contagious and spreads via respiratory droplets and direct contact [7]. Recent studies even suggest the potential for airborne spread in some circumstances [8,9]. At the time of this writing, neither the precise aerosol viral load nor the minimum infectious dose of SARS-CoV-2 has been identified [10], and the challenge for pandemic control is to limit the transmission of SARS-CoV-2 by asymptomatic or presymptomatic individuals [11]. Various protective barrier enclosures (PBEs) have been reported to reduce the aerosol risk during endoscopy, such as covering the patient’s upper body with a special box [12], using a face shield [13], or using a special mouthpiece [14]. Because of the COVID-19 pandemic, however, many HCWs are facing the problem of a shortage of PPE, including gowns, hairnets, and face shields/goggles [15,16]. Therefore, there is a need for a barrier that allows endoscopy to be performed without compromising the cleanliness of PPE from droplets and aerosols generated during endoscopy. The United States Food and Drug Administration warns that the use of PBEs without negative pressure may pose increased health risks during tracheal intubation in patients with COVID-19 [17]. We therefore developed a patient-covering negative-pressure box model for EGD that uses disposable vinyl films (Endo barrier^®^; Okura Industrial Co. Ltd., Kagawa, Japan) (EB) [18]. The new EB system has been produced to reduce aerosol and droplet diffusion during EGD.

The purpose of this study was to verify whether EB use during EGD effectively protects the examiner from exposure to aerosols and droplets using a particle counter and adenosine triphosphate (ATP) contamination tester.

## 2. Materials and Methods

### 2.1. Study Design, Setting, and Participants

This prospective, observational study was conducted at Kagawa Prefectural Shirotori Hospital from February 2021 to March 2021. Eighty consecutive unsedated patients who underwent screening EGD with EB use were prospectively enrolled. The inclusion criterion was an age of >18 years. The exclusion criteria were refusal to use the EB, a need for emergency endoscopic treatment, the presence of psychiatric disease or psychiatric symptoms, and the presence of severe respiratory disease. All EGD procedures were performed by a single endoscopist (S.F.) in the same room measuring 19.39 m^2^, with a total supply of three air changes per hour, internal recirculation at 23 °C ± 1 °C, and humidity of <60%.

### 2.2. Endo Barrier^®^

**Figure 1:**
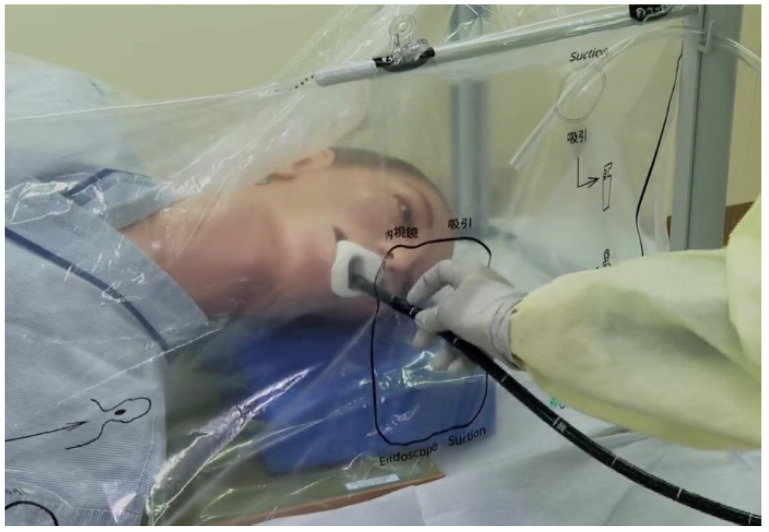
Patient-covering negative-pressure box (Endo barrier^®^).

The EB, a newly developed patient-covering negative-pressure box with disposable vinyl films for preventing droplet transmission, was used during EGD (Figure 1). The patient was placed in the left lateral position with his or her head covered by a disposable vinyl film. The facial side of this film has four access routes for the endoscope, suction tube, aspiration tube, and oxygen tube. At the patient’s lower side of the film, the endoscope was inserted into the box via a small hole opened by the endoscopist’s finger. At the patient’s upper side of the film, a continuous-suction tube under negative pressure of −20 kPa was inserted into the box to create a negative-pressure box. These access orifices were marked with black paint on the film. The endoscopists were positioned directly opposite the patient’s face.

### 2.3. Particle Measurement

**Figure 2:**
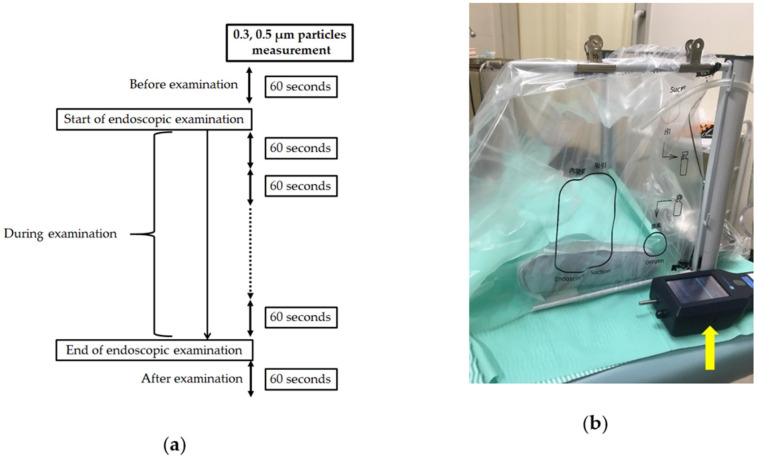
Particle measurement. (**a**) Examination flowchart. (**b**) Image of optical particle counter (yellow arrow) located in front of examiner at patient’s face side.

To assess the examiner’s exposure to aerosols, 0.3and 0.5µm particles (simulated aerosols) were measured before and after EGD by a handheld optical particle counter (Model 3889; Kanomax Japan, Osaka, Japan) that suctioned air at 2.83 L/min (Figure 2a). First, the particle counter was calibrated at least 30 min before the first procedure to maintain a stable baseline reading. Second, the instrument was usually placed within 10 cm from the patient’s mouth once the patient had entered the room and measured the air for at least 60 s before the start of the procedure. Third, the changes in aerosol levels were traced continuously every 60 s during EGD until 60 s after EGD (Figure 2b). The interunit precision was 50% ± 20% higher for 0.3 µm aerosols and 100% ± 10% higher for 0.5 µm aerosols compared with standard aerosols [19,20]. To minimize the influence of environmental factors on the aerosol readings, the following preliminary test was performed. A cutoff value of increased particles was configured by performing 10 measurements of the particles in the same setting not undergoing EGD (as a control group). As described in a previous report [20], the cutoff value was set at the mean ± 2 standard deviations (SDs) calculated from the 10 attempts. The mean ± 2SD of an increased ratio of 0.3 and 0.5 µm particles was 3.96 ± 14.41 and −0.14 ± 16.92, respectively (Supplementary Figure S1). Patients were classified as having increased aerosols when the values of the aerosols were more than the cutoff value for each particle size.

### 2.4. ATP Measurement

**Figure 3:**
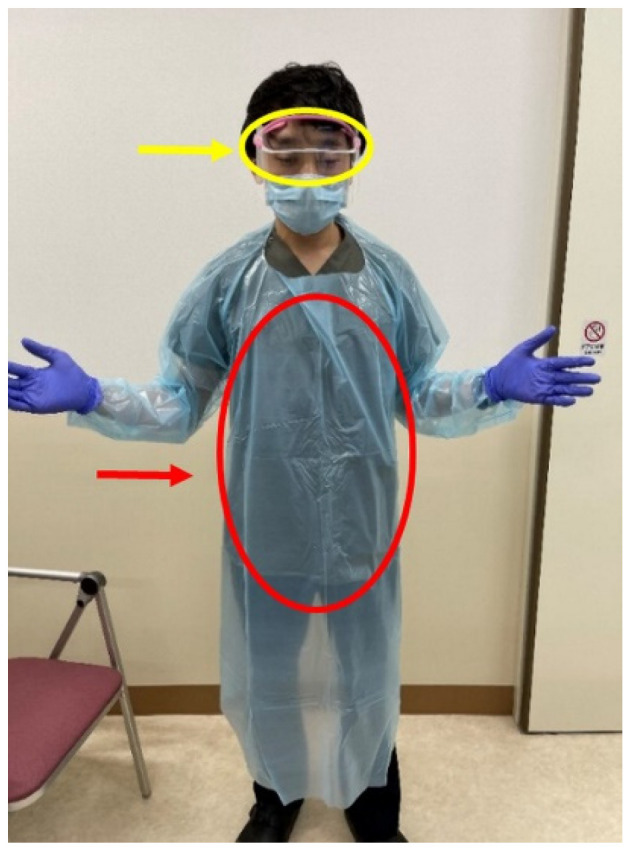
ATP measurement areas on goggles (yellow arrow and circle) and disposal vinyl gowns (red arrow and circle) using the swab technique.

Surfaces with potential ATP (simulated droplets) contamination of goggles and disposal vinyl gowns were evaluated before and after EGD using the LuciPac^®^ Pen and Lumitester PD-20^®^ System (Kikkoman Biochemifa Co., Tokyo, Japan) (Figure 3). ATP is a point-of-care test for acquiring immediately available results in relative light units (RLU), thus enabling measurement of the remnants of microorganisms and their protein components including viruses and proteins. The device detects concentrations that are expressed as the number of RLU. The LuciPac^®^ Pen is a kit that combines reagents with a swab used to pick up ATP from the test medium. The investigator (S.F.) took the swab from the kit, moistened it with sterilized water, uniformly wiped the object in every direction while avoiding the dry parts, returned the swab to the LuciPac^®^ Pen, shook it well to ensure a reaction with the luminescent reagent, and inserted the LuciPac^®^ Pen into the measurement chamber of the Lumitester PD-20^®^ apparatus. According to the manufacturer, the benchmark for detecting contamination on non-critical instruments using these devices ranges from 200 to 500 RLU, and that for detecting contamination on the dominant hand is ≤1000 RLU. Therefore, we considered the anesthetists’ working environment in the operation room and their dominant hand to be clean at benchmarks of ≤500 and ≤1000 RLU, respectively.

Standard goggles and disposal vinyl gowns were used before the EGD procedure. Each pair of goggles was cleaned with alcohol before every EGD procedure. We acquired ATP samples to compare the ATP level before EGD with that after EGD. These samples were obtained in the same manner using the swab technique from the whole area of the goggles and from the top to the bottom of the front area of the gowns.

### 2.5. Outcome Measures

The primary outcome was assessment of the changes in aerosols before and after EGD. The secondary outcome was analysis of the increase in the ratio of aerosols during EGD and examination of the changes in droplets using ATP before and after EGD. The cutoff value for the degree of contamination by the rapid ATP test was set at >150 RLU.

### 2.6. Statistical Analysis

Data are expressed as mean ± SD. All statistical analyses were performed using Prism 6 software (GraphPad Software, La Jolla, CA, USA). A Wilcoxon matched-pairs signed-rank test was used to evaluate statistical differences in the primary outcome measure. A nonparametric Wilcoxon/Mann–Whitney U-test was used to examine the statistical significance of differences between the two groups. A *p* value of <0.05 was considered significant.

## 3. Results

### 3.1. Participants and Descriptive Data

The baseline characteristics of all 80 patients are shown in Table 1. No patients met the exclusion criterion. The patients’ mean age was 65 ± 15.8 years, and 37 were male. Their mean body mass index was 22.4 ± 3.8 kg/m^2^, and 68.8% (55/80) of patients had a medical history. Transnasal endoscopy was performed in 60% (48/80) of patients, and oral endoscopy was performed in 40% (32/80). No patients received intravenous sedation. The mean examination time was 236.6 ± 77.2 s, and biopsies were performed in 11 (13.8%) patients. During endoscopic examination, 29 (36.3%) patients developed burping, 23 (28.8%) developed reflux vomiting, 20 (25.0%) developed coughing, 2 (2.5%) developed sneezing, and 5 (6.3%) exhibited body motion. Because of faulty measurement equipment during the examination, 7 of the 80 patients underwent inadequate testing with the particle counter and ATP measurements.

### 3.2. Outcome Data

**Figure 4:**
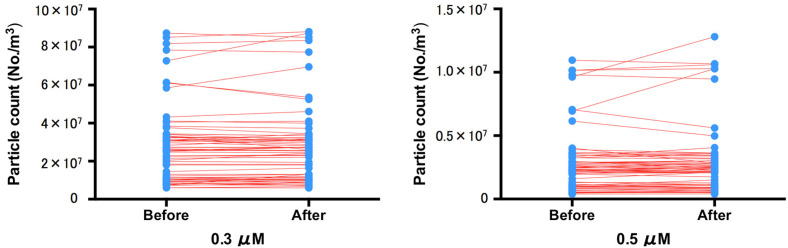
Particle counts of 0.3-, 0.5-, and 1.0-μm particles before and after EGD with the EB (*n* = 73). The 0.3-, 0.5-, and 1.0-μm particles did not increase in 95.8% (70/73), 94.5% (69/73), and 65.7% (48/73), of patients, respectively. When we analyzed the particle counts before and after EGD in each group, there were no significant differences in the production of 0.3-μm particles (*p* = 0.697), 0.5-μm particles (*p* = 0.926), or 1.0-μm particles (*p* = 0.515). The blue dotted line indicates the 95% confidence interval.

When we examined patients with and without increased aerosols before and after EGD, we found that 0.3- and 0.5-μm particles did not increase in 95.8% (70/73) and 94.5% (69/73) of patients, respectively. When we compared the total particle counts before and after EGD in each group, there was no significant difference in the production of either 0.3-μm particles (*p* = 0.697) or 0.5-μm particles (*p* = 0.926) (Figure 4).

**Figure 5:**
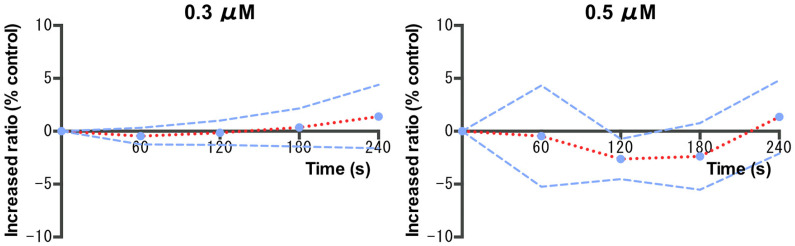
120, 180, and 240 s from the start of EGD with the EB (*n* = 63). The red line represents the mean particle count (0.3, 0.5, and 1.0 μm). A Wilcoxon matched-pairs signed-rank test was used to evaluate statistical differences. EGD = esophagogastroduodenoscopy; EB = Endo barrier^®^.

Of 73 patients, 63 who successfully underwent continuous measurement of particles every 60 s during EGD were analyzed. The reasons for excluding 10 patients were a short examination time (<180 s) and technical errors. The 63 patients had an examination time of >240 s, which corresponded to the mean examination time of 236.6 s in all 80 enrolled patients. Accordingly, the particles were measured at the four time points of 60, 120, 180, and 240 s when the start of EGD was defined as 0 s. The increased ratio of 0.3- and 0.5-μm particles during EGD was calculated as the baseline control when the start of EGD was calibrated as 0% (Figure 5). The mean ratio of 0.3-μm particle production was −0.4% ± 3.2% at 60 s, −0.15% ± 4.7% at 120 s, 0.36% ± 7.1% at 180 s, and 1.4% ± 12.3% at 240 s. The mean ratio of 0.5-μm particle production was −0.4% ± 18.8% at 60 s, −2.6% ± 7.5% at 120 s, −2.4% ± 12.0% at 180 s, and 1.4% ± 13.7% at 240 s.

The mean ATP level before EGD was 6.9 ± 6.8 RLU on goggles and 1.59 ± 19.9 RLU on gowns. The mean incremental difference in the ATP levels before and after EGD was −0.6 ± 16.6 RLU on goggles and 1.59 ± 19.9 RLU on gowns (Table 2). These ATP levels were within the cutoff value of 150 RLU.

**Figure 6:**
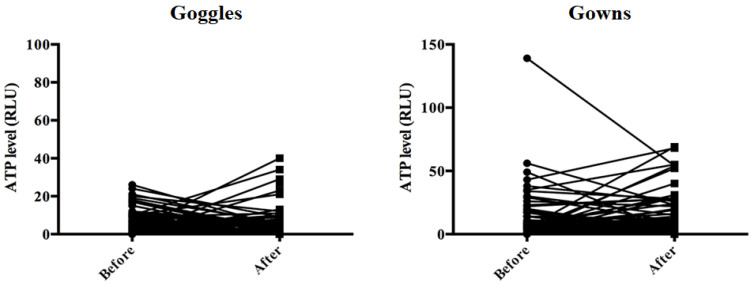
Changes in ATP concentrations on goggles and plastic gowns before and after EGD. ATP = adenosine triphosphate; EGD = esophagogastroduodenoscopy.

No significant contamination was found on either the goggles or gowns according to the ATP levels of all cases (*n* = 73) (Figure 6).

Seventy-three patients were divided into two groups: those with increased 0.3-μm and/or 0.5-μm aerosols (*n* = 6) and those without increased aerosols (*n* = 67). The possible associated factors were examined. No significant factors were detected in the comparison between patients with and without increased aerosols. No complications involving perforations, bleeding, and respiratory suppression occurred in any patients during the current study.

## 4. Discussion

To the best of our knowledge, this is the first study to analyze the effects of EB use during EGD. The present study revealed found two important clinical findings. First, the EB provided a certain protective effect against exposure of the examiners to aerosols and droplets. Second, the EB may reduce PPE contamination and allow the continued use of PPE. The EB is expected to be useful during the COVID-19 outbreak, especially when the supply of PPE is inadequate.

In a study from northern Italy, Repici et al. [21] conducted a telephone survey 2 weeks after endoscopy and found that only 1 of the 802 patients who responded showed positive results for COVID-19. Similarly, the SCOTS (Symptoms, infectious Contacts, Occupational risk, Travel risk, shielding status) project performed a prospective multicenter survey of 6208 endoscopic procedures from 18 United Kingdom centers during the pandemic recovery period and examined the endoscopy-associated risk of COVID-19 transmission. Telephone surveillance of patients at 1 and 2 weeks revealed no cases of COVID-19 among either patients or staff [22]. Taken together, the emerging evidence suggests that gastrointestinal endoscopy is relatively safe for patients and HCWs when strict infection prevention measures are taken. However, a major concern regarding COVID-19 is the presence of asymptomatic patients. The asymptomatic rate among infected individuals is estimated as 1.2% [23]. During pre-endoscopy screening, 3 of 2611 (0.11%; 95% confidence interval, 0.00–0.33%) asymptomatic patients showed positive results for SARS-CoV-2 on a nasopharyngeal swab [22]. In clinical practice, screening testing of all patients seems unrealistic. Thus, in addition to a COVID-minimized pathway (preprocedure questionnaire and PPE use) and control measures (air exchange and room cleaning) described in Western [4] and Asian [5] endoscopy position statements, further preventive measures to reduce virus transmission may be needed in EGD during the COVID-19 pandemic era. At present, several PBEs for shielding aerosol dispersal from patients are still under investigation [12,13,14,24]. Continuous dental suction in the oral cavity significantly diminished the counts of particles of all sizes during EGD, probably leading to reduction of room pollution [25]. The concept of EB use introduced in the present study is also based on negative-pressure suction to prevent diffusion of virus throughout the room.

A recent proof-of-concept study using laser particle counters showed that EGD was associated with increased levels of aerosol-sized particles [25]. Specifically, the increase in 0.3-, 0.5-, 0.7-, and 1.0-μm particles supports EGD as an aerosol-generating procedure. Our study used particle sizes of 0.3 and 0.5 μm, which are close to the size of SARS-CoV-2, and explored the efficacy of EB use during EGD. The 0.3- and 0.5-μm particles did not increase in 95.8% and 94.5% of patients, respectively, during EGD under EB use. There were also no significant differences in the production of 0.3- and 0.5-μm particles when compared with the total 0.3- and 0.5-μm particle counts before and after EGD. Consequently, EB use may help to minimize the examiner’s exposure to aerosol-associated virus transmission. Sagami et al. [20] used a particle counter to determine whether aerosols increased with or without endoscopy (control group) in the presence of a head-covering enclosure. The ratio of patients with increased aerosols between before and after EGD was significantly higher in the endoscopy group than in the control group [73.8% (76/103) vs. 22.2% (20/90), respectively], and the result of increased aerosol counts was similar. Notably, the proportion of patients with increased aerosols was 22.2% (20/90) in the control group without endoscopy. This may support the information that aerosols are easily influenced by various environmental factors including temperature, human movements, and air conditioning equipment. To minimize these environmental factors, we established a cutoff value of increased particles in the present study. However, infectious risk seems to be confined to particles of ≥0.5 μm; the risk of 0.3-μm aerosols, which approximate the size of SARS-CoV-2, remains unclear [26]. Because this is a complex subject with many variables, the evidence may be difficult to clarify.

Sagami et al. [20] reported that burping during EGD and the body mass index were risk factors for increased aerosols. In contrast, when we compared variables between patients with (*n* = 6) and without (*n* = 67) increased aerosols, no significant risk factors were detected as shown in Table 3. This may be explained by the small number of patients with increased aerosols.

Only a few studies have focused on droplet exposure among endoscopists using ATP Lumitesters. The World Health Organization adopts a 5-mm cutoff to distinguish between airborne transmission (<5 mm) and droplet transmission (>5 mm). Droplet transmission is defined as the transmission of diseases by expelled particles that have a propensity to settle quickly to the ground, usually within 1 m of the site of generation, because of their size. Thus, infection by droplet transmission occurs when the distance between infected patients and HCWs is small [26]. Therefore, precautions for endoscopists working close to patients must be extremely precise to prevent droplet transmission. According to a randomized trial using ATP measurement, diagnostic endoscopy did not contaminate the endoscopist’s face shield; however, the risk of contamination could be further reduced by placing the patient in the prone position [27]. In another study, involving measurement using fluorescent dyes, placing the patient at least 1 m below the top of the endoscopist’s head or using a barrier device during EGD minimized the endoscopist’s exposure to visible droplets on the face [28]. In the present study, when comparing changes in aerosol counts and ATP levels before and after EGD, no significant increase in contamination was found on either the goggles or gowns according to the measured ATP levels of all cases. This finding suggests a positive effect of the EB against droplet transmission. Based on the results of previous studies and the present study, the use of patient-covering PBEs would be an effective option for minimizing aerosol and droplet transmission.

The EB consists of two main components: disposable vinyl film and specially designed frame. Okura Industries Corporation has recently announced that the price of single special frame amounted for approximately USD 545 in Japan. The cost for single disposable vinyl film is about USD 1.82 per case. Unfortunately, these products are not covered by Japanese medical insurance system, so in many cases the hospital must own the payment for them, or the patients themselves must pay for the disposable vinyl film.

Our study has several limitations. First, this was an observational study with a single arm in a single institution. We were unable to establish a control group in which the EB was not used because the study begun during the pandemic and EB use was introduced as strict preventive measure in our hospital. Second, the total number of patients was relatively small, and the number of analyzed patients further decreased because of several technical and sampling errors. Third, this study involved a single endoscopist to minimize bias of the endoscopic technical procedure. Fourth, all included patients underwent EGD without sedation; thus, whether the presence or absence of sedation was associated with the increased aerosols remains uncertain. Fifth, small aerosols are easily dispersed because they are sensitive to ambient airflow, strong lateral currents, and ventilation [26]. Therefore, they cannot stay in a particular place for a long time. Aerosols may not be suitable for measurement because they are greatly affected by the airflow in the environment. However, we focused on endoscopists’ exposure to aerosols by placing the counter instrument between the patient’s oral cavity and the endoscopist and then measured the aerosol concentration.

In conclusion, EB use during EGD demonstrated an acceptable effect on aerosols and a sufficient outcome on droplets in terms of virus exposure among examiners.

## Figures and Tables

**Table 1:** Clinical data of patients who underwent EGD with EB.

	*n* = 80
Age, years	65 ± 15.8
Male sex	37 (46.3)
Body mass index, kg/m^2^	22.4 ± 3.8
Current smoking	2 (2.5)
Alcohol drinking (>120 g/week)	4 (5.0)
Medical history	55 (68.8)
Type of endoscope	
Nasal	48 (60.0)
Oral	32 (40.0)
Examination time, seconds	236.6 ± 77.2
Symptoms during EGD	
Burping	29 (36.3)
Reflex vomiting	23 (28.8)
Cough	20 (25.0)
Sneezing	2 (2.5)
Body motion	5 (6.3)
Interventions	
None	69 (86.2)
Biopsy	11 (13.8)

Data are presented as mean ± standard deviation or *n* (%). EGD = esophagogastroduodenoscopy; EB = Endo barrier^®^.

**Table 2:** Outcome results: Comparison of changes in aerosol counts and ATP levels before and after EGD.

Variables	*n* = 73
Patients with unincreased aerosols after vs. before endoscopy	
0.3 μm	70/73 (95.8)
0.5 μm	69/73 (94.5)
Change in increased aerosols after vs. before endoscopy, ×10^6^/m^3^	
0.3 μm	0.77 ± 4.7
0.5 μm	−0.37 ± 4.7
Change in ATP on goggles between before and after EGD, RLU	−0.6 ± 16.6
Change in ATP on gowns between before and after EGD, RLU	1.59 ± 19.9

Data are presented as *n* (%) or mean ± standard deviation. ATP = adenosine triphosphate; EGD = esophagogastroduodenoscopy.

**Table 3:** Patients’ baseline characteristics and comparison of procedure-related factors between patients with and without increased aerosols during EGD with the EB.

	Patients with Increased Aerosols, *n* = 6	Patients without Increased Aerosols, *n* = 67	*p* Value
Age, years	63.0 ± 18.8	66.0 ± 15.6	0.74 *
Male sex	2 (33.3)	30 (44.8)	0.69
Body mass index, kg/m^2^	23.0 ± 2.5	22.4 ± 4.0	0.86 *
Current smoking	1 (16.7)	1 (1.5)	0.16
Alcohol drinking	0 (0.0)	3 (4.5)	1
Medical history	2 (33.3)	47 (70.2)	0.09
Nasal endoscope	5 (83.3)	37 (55.2)	0.23
Oral endoscope	1 (16.7)	33 (44.8)	0.23
Examination time, seconds	206.7 ± 42.4	240.7 ± 81.4	0.96 *
Cough	1 (16.7)	18 (26.9)	1
Reflux vomiting	2 (33.3)	20 (29.9)	1
Sneezing	0 (0.0)	2 (3.0)	1
Burping	2 (33.3)	25 (37.3)	1
Body movement	0 (0.0)	5 (7.5)	1
Interventions	0 (0.0)	10 (14.9)	0.59

Data are presented as mean ± standard deviation or *n* (%). Data were assessed with Fisher’s exact test or * Mann–Whitney U-test. EGD = esophagogastroduodenoscopy; EB = Endo barrier^®^.

## Data Availability

Not appliable.

## Supplementary Materials

The following are available online at https://www.mdpi.com/article/10.3390/diagnostics11091679/s1.
