# Supplementary material for: Clinical Efficacy of Novel Patient-Covering Negative-Pressure Box for Shielding Virus Transmission during Esophagogastroduodenoscopy: A Prospective Observational Study
Source: Diagnostics (Basel). 2021 Sep 14;11(9):1679. doi: 10.3390/diagnostics11091679 (PMC8470820; doi:10.3390/diagnostics11091679)

Figure S1. To minimize the influence of environmental factors on the aerosol readings, a cutoff value of increased particles was configured by performing 10 particle measurements in the setting without EGD. The mean cutoff value ± 2SD of an increased ratio of 0.3- and 0.5-μm particles was 3.96 ± 14.41 and −0.14 ± 16.92, respectively. Based on these data, increased aerosols were defined as an aerosol reading of more than the cutoff value for each size of particles. EGD = esophagogastroduodenoscopy; SD = standard deviation.

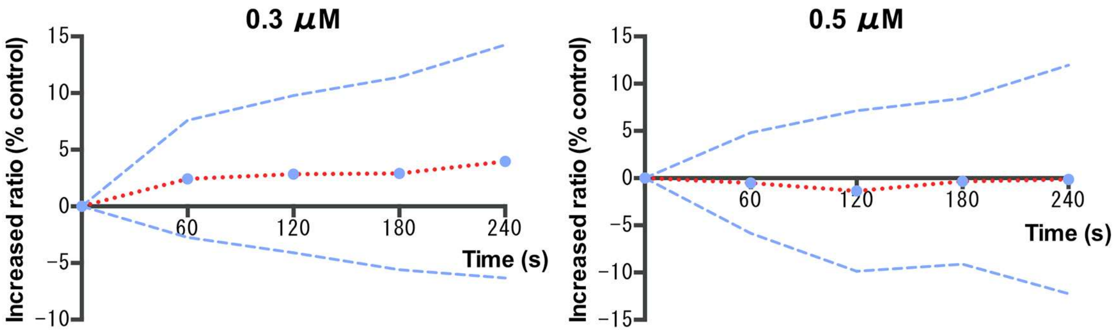

Supplement: Supplementary file 1 [file diagnostics-11-01679-s001.zip › diagnostics-1358700-supplementary.pdf]
